# Weight statistics controls dynamics in recurrent neural networks

**DOI:** 10.1371/journal.pone.0214541

**Published:** 2019-04-09

**Authors:** Patrick Krauss, Marc Schuster, Verena Dietrich, Achim Schilling, Holger Schulze, Claus Metzner

**Affiliations:** 1 Cognitive Computational Neuroscience Group at the Chair of English Philology and Linguistics, Department of English and American Studies, Friedrich-Alexander University Erlangen-Nürnberg (FAU), Erlangen, Germany; 2 Experimental Otolaryngology, Neuroscience Group, University Hospital Erlangen, Friedrich-Alexander University Erlangen-Nürnberg (FAU), Erlangen, Germany; 3 Biophysics Group, Department of Physics, Friedrich-Alexander University Erlangen-Nürnberg (FAU), Erlangen, Germany; Plymouth University, UNITED KINGDOM

## Abstract

Recurrent neural networks are complex non-linear systems, capable of ongoing activity in the absence of driving inputs. The dynamical properties of these systems, in particular their long-time attractor states, are determined on the microscopic level by the connection strengths *w*_*ij*_ between the individual neurons. However, little is known to which extent network dynamics is tunable on a more coarse-grained level by the *statistical* features of the weight matrix. In this work, we investigate the dynamics of recurrent networks of Boltzmann neurons. In particular we study the impact of three statistical parameters: *density* (the fraction of non-zero connections), *balance* (the ratio of excitatory to inhibitory connections), and *symmetry* (the fraction of neuron pairs with *w*_*ij*_ = *w*_*ji*_). By computing a ‘phase diagram’ of network dynamics, we find that balance is the essential control parameter: Its gradual increase from negative to positive values drives the system from oscillatory behavior into a chaotic regime, and eventually into stationary fixed points. Only directly at the border of the chaotic regime do the neural networks display rich but regular dynamics, thus enabling actual information processing. These results suggest that the brain, too, is fine-tuned to the ‘edge of chaos’ by assuring a proper balance between excitatory and inhibitory neural connections.

## Introduction

In contrast to the artificial neural networks used in deep learning, which typically have a strict feed-forward structure, the networks of the brain contain many loops and are therefore recurrent in nature. This feature allows the cortex to maintain dynamical activity even without incoming external stimuli [1] and may therefore underlie such diverse operations as short-term memory [2–4], the modulation of neuronal excitability with attention [2, 5, 6], or the generation of spontaneous activity during sleep [7–9].

The relation between structure and function in recurrent neural networks is a topic of considerable interest in the neurosciences and has already been addressed in several important publications. In 1988, a transition from a stationary phase to a chaotic phase was predicted [10], occurring at a critical value of a ‘gain’ parameter that controls the width of the neuron’s sigmoidal function. In 2007, it was shown that Network structure of the cerebral cortex shapes functional connectivity on multiple time scales [11]. A paper from 2011 studied the effect of broad degree distributions on network dynamics by interpolating between a binomial and a truncated power-law distribution for the in-degree and out-degree of the neurons independently [12]. In 2014, it was demonstrated that (1) for homogeneous external input, the structure of pairwise correlations between neuron states is mainly determined by the local recurrent connectivity, that (2) homogeneous external inputs provide an additive, unspecific contribution to the correlations, that (3) inhibitory feedback effectively decorrelates neuronal activity, even if neurons receive identical external inputs, and, finally, that (4) identical synaptic input statistics to excitatory and to inhibitory cells increases intrinsically generated fluctuations and pairwise correlations [13]. In 2016, a paper analyzed the anatomical origins of oscillations in the cortical microcircuit. Based on a theoretical reduction of network dynamics, a sensitivity measure was derived, resulting in a frequency-dependent connectivity map that revealed connections crucial for the peak amplitude and frequency of the observed oscillations and identifies the minimal circuit generating a given frequency [14].

Recently, more sophisticated statistical properties of weight matrices were explored. For instance, a model introduced a structured component of connectivity, in addition to random connections, which effectively embeds a feed-forward structure via unidirectional coupling between a pair of orthogonal modes [15]. Another approach studied a class of recurrent network models in which the connectivity is a sum of a random part and a minimal, low-dimensional structure. It was shown that, in such networks, the dynamics is low dimensional and can be directly inferred from connectivity using a geometrical approach [16]. Still another approach focused on the eigenvalue structure of the weight matrix and identified structural properties of networks that are associated with non-normality [17]. A more coarse-grained approach showed that coupling among cortical modules is central. The highest dynamical richness of the network emerges at a critical connectivity at the verge of physical disconnection. Stronger coupling leads to a persistently coherent activity among the modules, while weaker coupling precipitates the activity to be localized solely within the modules [18]. Finally, the effect of external inputs on network dynamics was explored. A paper identified a general criterion that distinguishes two classes of networks depending on properties of the connectivity matrix: networks in which all inputs lead to weak, decaying transients, and networks in which specific inputs elicit strongly amplified transient responses and are mapped onto orthogonal output states during the dynamics [19].

Recent micro-anatomical studies of the brain revealed that neural connectivity in the mammalian cortex has unique statistical properties. In particular, it was found that connections are sparse (low density), so that only a small fraction of possible connections are realized. The distribution of connection strengths is close to log-normal, and thus highly skewed, with a fat tail towards large magnitudes [20, 21]. Although the total number of non-zero connections can vary strongly between neurons, the ratio of excitatory to inhibitory connections is relatively constant [22]. Moreover, cortical networks contain a ‘skeleton’ of strongly connected neurons, linked pairwise in a bidirectional, symmetric way. This skeleton is embedded in a ‘sea’ of more weakly, non-symmetrically connected neurons [20].

Whereas the role of this peculiar connection structure is still poorly understood, certain features seem to affect whether the brain can properly act as an information processor. For example, it has been shown that recurrent neural networks can show chaotic behavior for certain ratios between excitatory and inhibitory connections [1, 23]. It has even been speculated that certain social dysfunctions, such as autism and schizophrenia, are related to an elevated cortical excitation/inhibition balance [24]. Moreover, the discovered skeleton of neurons with strong bi-directional links may help to optimize information storage [25].

In a recent paper [26], we have investigated the relation between connectivity and system dynamics in small motifs of probabilistic neurons with binary outputs, assuming discrete, ternary connection strengths. We found that the balance between excitatory and inhibitory connections has a strong effect on the transition probabilities between successive motif states, whereas the total density of non-zero connections is less important.

Here, we extent our study to larger recurrent networks that consist of deterministic neurons with continuous outputs. Connection strength follow a random, log-normal weight distribution, but have prescribed values of the three control parameters density, balance, and symmetry. We analyze how these parameters affect the dynamical properties of the networks, in particular the Lyapunov exponent of the system trajectory in state space, the period length of cyclic attractors, and the cross correlation between individual neuron states.

As has been previously shown by Hopfield [27], networks with a very large fraction of symmetric bidirectional connections (symmetry parameter close to one) tend to end up in stationary fixed points. We therefore focus on moderate and small symmetry parameters, and explore the two-dimensional phase diagram of system dynamics as a function of balance and density.

We find that this two-dimensional phase plane consists of three basic regions, corresponding to the possible attractors in deterministic and autonomous dynamical systems: periodic state cycles, chaos, and stationary fixed point behavior. Strikingly, it is almost exclusively the balance parameter that controls in which of these three regimes a neural network is located, while the overall density of connections has a much weaker influence. In particular, the networks behave in a way that is suitable for information processing purposes only in a narrow range of balance parameters, located at the edge of the chaotic phase. This theoretical result is in line with the experimental finding that neural networks in the mammalian cortex have moderate degrees of symmetry and are tuned to rather specific values of balance, whereas connection density can vary widely between neurons and over time.

## Methods

### Neural network model

Our neural networks are based on simple deterministic neurons with zero bias (zero threshold). The total input *z*_*i*_(*t*) of neuron *i* at time *t* is calculated as:

$$\begin{array}{c} z_{i}\left(t\right)=\sum_{j=1}^{n}w_{ij}\;y_{j}\left(t-1\right)\; \end{array}$$ (1)

where *y*_*j*_(*t* − 1) is the state of neuron *j* at time *t* − 1 and *w*_*ij*_ is the connection weight from neuron *j* to neuron *i*. The new state *y*_*i*_(*t*) of neuron *i* is computed as

$$\begin{array}{c} y_{i}\left(t\right)=\frac{1}{1\;+\;e^{-z_{i}\left(t\right)}}. \end{array}$$ (2)

When simulating the dynamics of the networks, all neurons are updated simultaneously. The total state of a neural network at time step *t* can be summarized by the *n*-dimensional vector $\vec{y}\left(t\right)=(y_{1}\left(t\right),\ldots ,y_{n}\left(t\right))$, where *y*_*i*_(*t*) is the output state of neuron *i* at this time. In each particular case, we simulated *T*_*max*_ = 10^6^ time steps.

### Random weight matrix

The structure of a given neural network is defined by its weight matrix *W* = {*w*_*ij*_}. Here, we consider networks in which self-connections are forbidden, so that *w*_*ii*_ = 0. For all non-zero matrix elements, the magnitudes of the weights are distributed according to a log-normal distribution,

$$\begin{array}{c} p\left(w_{ij}\right)\propto \text{lognormal}\left(\mu ,\sigma \right) \end{array}$$ (3)

with location *μ* and scale *σ*.

### Statistical control parameters $\hat{d},\hat{b},\hat{s}$

For a network with *n* neurons, the weight matrix has dimensions *n* × *n*. Due to the excluded self-connections, the diagonal elements of this matrix are zero, leaving a maximum possible number *n*(*n* − 1) of non-zero matrix elements. We denote the actual number of non-zero weights by *m* = *m*_+_ + *m*_−_, where *m*_+_ and *m*_−_ are the numbers of positive and negative weights, respectively. Furthermore, we denote the number of non-zero matrix elements *w*_*ij*_ for which a symmetric reverse connection *w*_*ji*_ = *w*_*ij*_ exists by *m*_*s*_. Based on these numbers, we define the *density* parameter $\hat{d}$, which varies between 0 for an unconnected and 1 for a fully connected network, by

$$\begin{array}{c} \hat{d}=\frac{m}{n(n-1)}. \end{array}$$ (4)

The *balance* parameter $\hat{b}$, which varies between −1 for a purely inhibitory and +1 for a purely excitatory connection matrix, is defined by

$$\begin{array}{c} \hat{b}=\frac{m_{+}-m_{-}}{m}. \end{array}$$ (5)

The *symmetry* parameter $\hat{s}$, which varies between 0 for a completely non-symmetric and +1 for a completely symmetric (Hopfield-like [27]) network, is defined by

$$\begin{array}{c} \hat{s}=\frac{m_{s}}{m}. \end{array}$$ (6)

**Fig 1:**
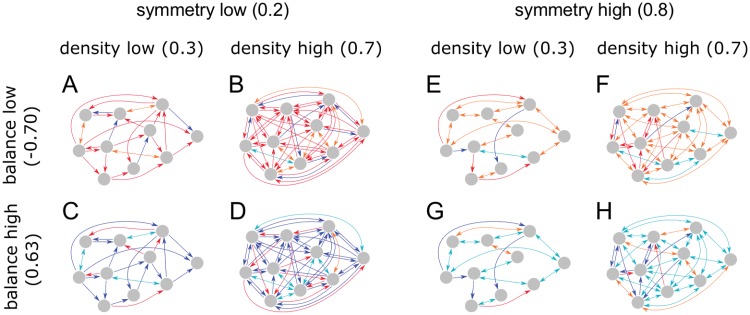
Visualization of the control parameters density $\hat{d}$, balance $\hat{b}$, and symmetry $\hat{s}$ for eight example networks (A-H). Neurons are represented by gray circles, non-zero connections between neurons by arrows. One-headed arrows stand for uni-directional, two-headed arrows for bi-directional connections. Blue/magenta connections are excitatory (*w*_*ij*_ > 0), red/orange connections inhibitory (*w*_*ij*_ < 0).

The meaning of these three control parameters is visualized in Fig 1.

### Generation of weight matrices

Random weight matrices with prescribed values of the parameters $\hat{d}$, $\hat{b}$, and $\hat{s}$ were generated in a series of steps. First, a fraction $\hat{d}$ of the weights were drawn independently from a log-normal distribution with location *μ* = 0 and scale *σ* = 1, whereas all remaining weights were set to zero. Second, in order to introduce inhibitory connections to the network, a fraction $(1-\hat{b})/2$ of the non-zero-weights were randomly selected and multiplied by −1. For all cases with symmetry $\hat{s}=0$, this produced already the final weight matrix.

For the case $\hat{s}>0$, further steps were required: the weights above the diagonal of the weight matrix were copied to below the diagonal, thereby creating a perfectly symmetric matrix. Finally, pairs of matrix elements below the diagonal were randomly selected and swapped iteratively, until the desired degree of symmetry $\hat{s}$ was achieved.

### Fraction of positive Lyapunov exponents *f*_λ>0_

Computing the new network state $\vec{y}\left(t\right)$ from the previous state $\vec{y}\left(t\;-\;1\right)$ can be formally described by a vectorial update function

$$\begin{array}{c} \vec{y}\left(t\right)=\vec{F}(\vec{y}\left(t-1\right)). \end{array}$$ (7)

Starting from an infinitesimally close initial state ${\vec{y}}_{*}\left(t\;-\;1\right)=\vec{y}\left(t\;-\;1\right)+\vec{\epsilon }$ will lead to a different final state ${\vec{y}}_{*}\left(t\right)=\vec{F}({\vec{y}}_{*}\left(t\;-\;1\right))$. The sensitivity of the update function to this infinitesimal perturbation can be measured by the differential quotient

$$\begin{array}{c} {\vec{F}}^{\;\prime }\left(\vec{y}\left(t\right)\right)\approx \frac{{\vec{y}}_{*}\left(t\right)-\vec{y}\left(t\right)}{|\vec{\epsilon }|}. \end{array}$$ (8)

The *maximum Lyapunov coefficient* λ of the update function is defined as

$$\begin{array}{c} \lambda =\langle \ln|{\vec{F}}^{\;\prime }\left(\vec{y}\left(t\right)\right)|\rangle_{t}, \end{array}$$ (9)

where 〈〉_*t*_ denotes the time average over all successive states of the system. It can be computed using well-established algorithms [28, 29]. A positive Lyapunov coefficient λ > 0 indicates that two nearby points in state space diverge exponentially, thus leading to irregular (chaotic) behavior. A zero or negative λ ≤ 0 indicates regular behavior. In general, within an ensemble of networks that are all characterized by the same set of control parameters $\left(\hat{d},\hat{b},\hat{s}\right)$, one finds λ-values of both signs. We therefore counted the fraction of networks (within the statistical ensemble of the considered parameter combination) that had a positive Lyapunov exponent. This fraction *f*_λ>0_ is represented as a color code and shown for all paramater combinations in the ‘phase diagrams’ below. Note that, the so-defined quantity *f*_λ>0_ reveals a transition at around 50%.

### Average period length *T*_av_

Our recurrent networks are deterministic and autonomous dynamical systems. Thus, their trajectory $\vec{y}\left(t\right)$ through *n*-dimensional state space is eventually governed by one of three possible attractors: a stationary fixed point, a cycle of period *T*, or chaotic behavior. For each investigated network, we characterize the type of attractor by the measured period length *T*, that is, the number of time steps before the system state repeats itself for the first time ($\vec{y}\left(t\right)=\vec{y}\left(t+T\right)$). This includes the special cases *T* = 1, corresponding to a stationary fixed point, and *T* = ∞, corresponding to a chaotic attractor. To identify repeating system states, we make use of a hash table. Since period lengths fluctuate for different networks from the same ensemble $\left(\hat{d},\hat{b},\hat{s}\right)$, we compute the ensemble average *T*_av_ and use this average for color coding the phase diagrams.

### Root mean square of cross correlations *ρ*_rms_

The Lyapunov coefficient λ and the period length *T* characterize the long-time behavior of the neural networks. Another property that is relevant for a network’s information processing ability is the degree of correlation between individual neuron states *y*_*i*_(*t*) at the same time step *t*. For each pair *i*, *j* of neurons, it can be quantified by the Pearson cross correlation coefficient, defined as

$$\begin{array}{c} \rho_{i,j}=\frac{{\left\langle (y_{i}\left(t\right)-{\bar{y}}_{i})(y_{j}\left(t\right)-{\bar{y}}_{j})\right\rangle }_{t}}{\sigma_{i}\sigma_{j}}, \end{array}$$ (10)

where ${\bar{y}}_{k}$ is the temporal mean of the time series *y*_*k*_(*t*) and *σ*_*k*_ its standard deviation. In cases where *σ*_*i*_ or *σ*_*j*_ were zero, *ρ*_*i*,*j*_ was set to 1. To characterize the global degree of correlation in a given neural network (without caring about the sign of the individual *ρ*_*ij*_), we computed the root mean square (RMS) over all neuron pairs

$$\begin{array}{c} \rho_{\text{rms}}=\sqrt{\frac{1}{n^{2}}\sum_{i,j}{\left(\rho_{i,j}\right)}^{2}}. \end{array}$$ (11)

This quantity was additionally averaged over all members of a given $\left(\hat{d},\hat{b},\hat{s}\right)$ ensemble and then used for color coding the phase diagrams.

## Results

We first consider non-symmetric networks ($\hat{s}=0$), that is, networks without any bidirectional links of exactly the same strength. For each combination of balance $\hat{b}$ and density $\hat{d}$ on a 11 × 10 regular grid, we generate an ensemble of 100 random networks. We then simulate the temporal dynamics of these networks, starting from random initial states. For each ensemble, we compute the fraction of positive Lyapunov coefficients *f*_λ>0_, the average period length *T*_av_, and the RMS of cross correlations *ρ*_rms_. The dependence of these dynamical quantities on the statistical control parameters is presented in the form of heat maps, which can be interpreted as dynamical ‘phase diagrams’ of these recurrent neural networks.

**Fig 2:**
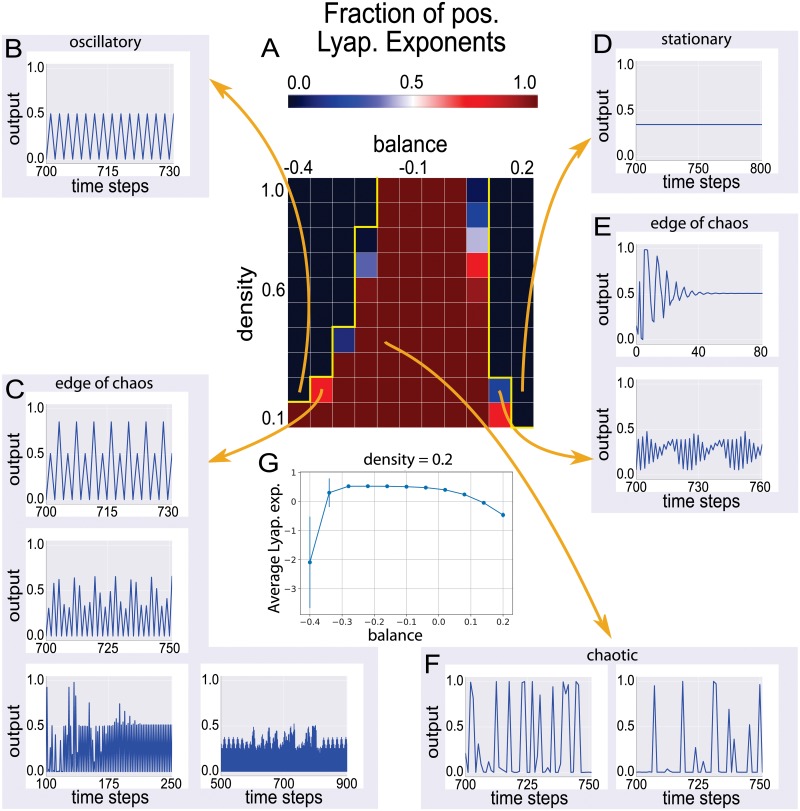
Dynamical phases in recurrent neural networks and characteristic output signals of individual neurons. (A) Two-dimensional phase diagram, showing the fraction of positive Lyapunov exponents $f_{\lambda >0}\;\left(\;\hat{b},\hat{d}\;|\;\hat{s}\;=\;0\;\right)$ as a function of the control parameters balance and density, for a constant symmetry parameter $\hat{s}\;=\;0$ (Note that in part G, we show the average Lyapunov exponent along a a one-dimensional cut through the 2D phase space of balance and density, for constant density 0.2). In the heat map, dark blue colors indicate *f*_λ>0_ ≈ 0, dark red colors *f*_λ>0_ ≈ 1. The red region in the center of the phase diagram is the chaotic regime, consistent with the irregular outputs of selected neurons (F). The ‘left’ blue region at negative balance values is the regime of cyclic attractors, often with small period lengths *T* ≈ 2, as demonstrated with the neuron output (B). The ‘right’ blue region at positive balance values is the regime of fixed points, as exemplified with the constant neuron output (D). Note that, in both cases (B, D) the fraction of positive Lyapunov exponents is zero since the dynamics is non-chaotic, and hence the color coding is identical in both cases. The most interesting dynamics is found at the edges of the chaotic regime (C, E), where one finds cases of periodic behavior with large period length *T* > 2, periodic behavior with intermittent bursts, decaying oscillatory behavior, and ‘beating’ oscillatory behavior. Note that, the sampled time traces depicted in the figure are from selected neurons, not necessarily from within the same network.

We initially focus on small networks of 100 neurons. When keeping the density close to one and gradually increasing the balance from negative to positive values, we find that the fraction of positive Lyapunov coefficients *f*_λ>0_, indicating chaotic behavior, is close to zero, except for a narrow interval of balance values around $\hat{b}\approx 0.1$. This chaotic interval broadens as the density parameter is reduced (Fig 2A). In the 2D phase diagram, the chaotic regime therefore has an approximately triangular shape.

Inspecting the temporal output signals of selected neurons in the investigated networks (Fig 2B–2G), it turns out that the two regimes with *f*_λ>0_ ≈ 0 at the ‘left’ and ‘right’ side of the chaotic regime correspond to periodic attractors (Fig 2B) and fixed point attractors (Fig 2D), respectively. The most interesting dynamics is found at the edge of the chaotic regime (Fig 2C and 2E), where one finds cases of periodic behavior with large period length *T* > 2, periodic behavior with intermittent bursts, decaying oscillatory behavior, and ‘beating’ oscillatory behavior.

**Fig 3:**
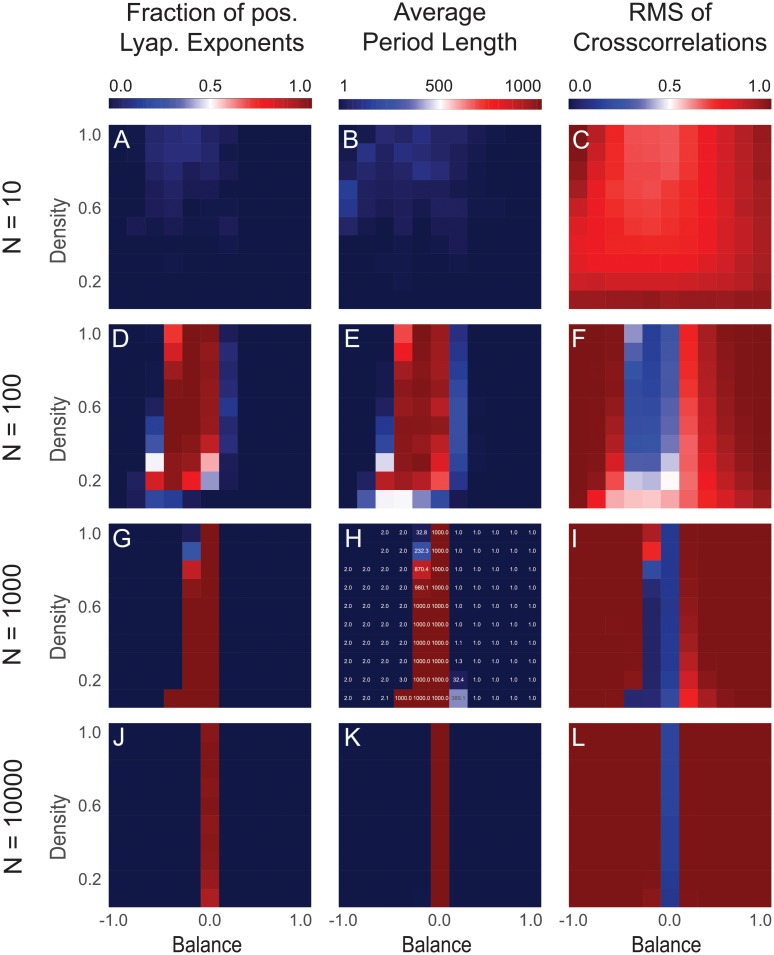
Comparing different dynamical measures, and the effect of system size. The columns correspond to the quantities *f*_λ>0_ (left), *T*_av_ (middle) and *ρ*_rms_ (right), as defined in the methods section. The rows from top to bottom correspond to increasing system sizes, characterized by the number of neurons *N* in the neural networks. For each of the 12 cases, a two-dimensional phase diagram is shown as a function of balance and density, keeping a constant symmetry parameter of $\hat{s}=0$. The three dynamic phases become apparent only for systems with a minimum size of *N* ≥ 100. The three different dynamical measures are mutually consistent. In particular, the chaotic regime is characterized by a *f*_λ>0_ close to one, by a diverging *T*_av_, and by a vanishing *ρ*_rms_. For large systems with *N* ≥ 10000, the density parameter has no more effect on the system dynamics, which is then controlled by the balance only.

In a next step, we compare the phase distribution of *f*_λ>0_ with that of the other two dynamical quantities (middle and right column in Fig 3). At the same time, we investigate the effect of system size (rows in Fig 3, with different numbers of neurons *N*).

We find that the three different dynamical quantities are mutually consistent. In particular, the chaotic regime is characterized by *f*_λ>0_ ≈ 1, by a diverging *T*_av_, and by a vanishing *ρ*_RMS_. The periodic regime is characterized by *T*_av_ ≈ 2 and by a relatively large *ρ*_rms_. The fixed point regime is characterized by *T*_av_ = 1 and, again, by a relatively large *ρ*_rms_. Approaching the chaotic regime from either side by changing the balance parameter, *T*_av_ is rapidly increasing in the border region.

With increasing system size, the influence of the density parameter on the dynamical phase of the networks is diminishing. For large networks with *N* ≥ 1000 neurons, the network dynamics is exclusively controlled by the balance parameter.

**Fig 4:**
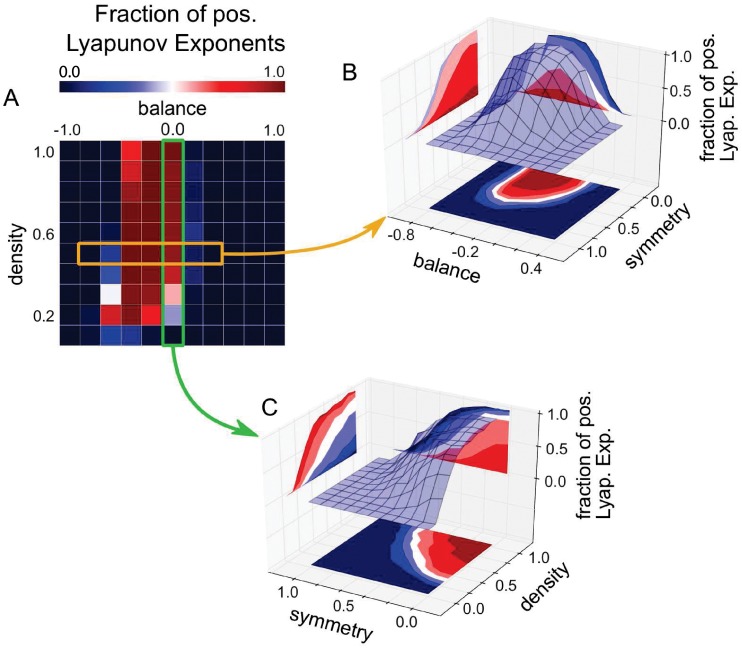
Effect of symmetry $\hat{s}$ on system dynamics. (A): Standard plot of *f*_λ>0_ as a function of balance and density, for constant symmetry $\hat{s}=0$. (B): Plot of *f*_λ>0_ as a function of balance and symmetry, for constant density $\hat{d}=0.5$ (see orange box in (A)). (C): Plot of *f*_λ>0_ as a function of symmetry and density, for constant balance $\hat{b}=0$ (see green box in (A)). For too large symmetry $\hat{s}\approx 1$, the system ends up in fixed point attractors, irrespective of balance and density. Note that the phase diagram shown in (A) is the same as shown in Fig 3D.

Finally, we investigate the effect of the symmetry parameter on the network dynamics (Fig 4). By computing a complete 3D phase diagram of *f*_λ>0_ as a function of all three statistical control parameters, we find that balance and density have only an effect on the system dynamics when the symmetry is smaller than one, that is, when there are sufficiently many non-symmetric connections between the neurons. For a too large symmetry $\hat{s}\approx 1$, the system ends up in fixed point attractors, irrespective of balance and density.

## Conclusion

One of the earliest studies [10] dealing with dynamical regimes in recurrent neural networks found that a transition from regular to chaotic behavior can be induced by increasing the nonlinearity of the neuron’s sigmoidal function. In this work, we have demonstrated that the dynamical behaviour of recurrent neural networks can be effectively tuned by certain statistical properties of the network’s connection weight matrix.

In particular, a large fraction of symmetric, bi-directional neural connections ($\hat{s}\approx 1$) favors fixed point attractors, and may therefore be useful for pattern completion tasks, as in the Hopfield model [27]. However, rich dynamical behavior is only possible for moderate or small degrees of symmetry. We point out, that besides our definition of the symmetry parameter, there are alternative definitions which are more fine grained. For instance, the symmetry definition by Esposito et al. [30] takes into account the magnitudes of the forward and backward connections, and vanishes in the case when only uni-directional connections exist. In addition, future extensions of our work could improve our present method to generate weight matrices with pre-defined symmetry, as the present method may create spurious correlations of weight magnitudes between the upper and lower triangle.

For non-symmetric networks, the statistical parameter with the largest impact on system dynamics is the balance $\hat{b}$ [26]. This ratio between excitatory and inhibitory connections controls, with high fidelity, whether a free-running neural network will behave stationary, oscillatory, or irregularly. Moreover, fine tuning of the balance parameter can bring the system to the edge of the chaotic regime, where the outputs of the neurons produce complex wave forms, and where the system may depend sensibly, but still regularly, on external inputs. We speculate that this regime is most suitable for purposes of neural information processing [31–35], and that biological brains may therefore control the parameter $\hat{b}$ in a homeostatic way [1, 36, 37].

By contrast, the impact of the overall connection density $\hat{d}$ on network dynamics, at least in realistically large systems with many neurons, is much smaller than that of the balance $\hat{b}$. In principle, a recurrent neural network can gain or loose a large random fraction of neural connections without changing its dynamical attractor state, as long as the balance $\hat{b}$ remains unchanged. This surprising robustness, for which the term graceful degradation has been coined [38], may help to keep the cortex functional during periods of growth and decay.

In this work we abstracted from biological detail in that each given neuron could have both, positive and negative output weights. By contrast, in the human brain the vast majority of neurons is either purely excitatory or purely inhibitory (Dale’s principle), although there are prominent exceptions to this rule, such as the dopaminergic transmission within the basal ganglia [39]. Nevertheless, it might be worthwhile to explore the impact of Dale’s principle on networks dynamics.

Future work will also need to clarify how recurrent neural networks, statistically tuned into specific attractor states, react to external inputs. A particularly interesting question will be whether the edge of chaos is also marked by a large mutual information between input signals and the internal sequence of states within the recurrent neural network. Furthermore, Wernecke et al. [40] proposed a method to test for partially predictable chaos, which might be applied to derive a more fine-grained description of the chaotic regime, including the edges of chaos.
